# Case report: Pediatric patient with severe clinical course of CTLA-4 insufficiency treated with HSCT

**DOI:** 10.3389/fimmu.2024.1484467

**Published:** 2024-11-18

**Authors:** Katarzyna Drabko, Julia Zarychta, Adrian Kowalczyk, Magdalena Cienkusz

**Affiliations:** ^1^ Department of Pediatric Hematology, Oncology and Transplantology, Medical University of Lublin, Lublin, Poland; ^2^ Student Scientific Society of Department of Pediatric Hematology, Oncology and Transplantology, Medical University of Lublin, Lublin, Poland

**Keywords:** CTLA-4 insufficiency, inborn errors of immunity, HSCT, pediatric patient, case report

## Abstract

**Background:**

Cytotoxic T lymphocyte–associated antigen-4 (CTLA-4) insufficiency is a rare disease belonging to inborn errors of immunity. Most cases of patients with CTLA-4 insufficiency are diagnosed in adults, therefore it is not a common problem in the clinical practice of pediatricians. However, it is worth noticing that most cases described in the literature show the first symptoms of the disease before the age of 18, but the phenotypic variability of patients complicates and delays the diagnostic process.

**Case description:**

Herein, we report a case of an almost 4-year-old patient whose first symptom of CTLA-4 insufficiency was thrombocytopenia after an upper respiratory tract infection, suggesting the diagnosis of primary immune thrombocytopenia, often occurring in pediatric patients. Due to the addition of symptoms suggesting a proliferative disease in this patient (pancytopenia, enlargement of lymph nodes, liver and spleen), a bone marrow biopsy was performed 11 months later, which did not confirm a hematopoietic tumor. Two years after the first symptoms appeared, the patient was referred for next-generation sequencing genetic testing, which confirmed the presence of a pathological CTLA-4 variant (c.356T>C). Due to the patient’s lack of response to the pharmacological treatment and the intensification of autoimmune symptoms that threatened the patient’s life, the patient underwent hematopoietic stem cell transplantation (HSCT) 34 months after the first occurrence of symptoms. After HSCT, the patient is alive and does not present any symptoms of autoimmunity.

**Conclusions:**

The first symptoms of some diseases classified as inborn errors of immunity are non-specific and may delay the final diagnosis. Therefore, it seems extremely important that practicing pediatricians should take into account inborn errors of immunity in the differential diagnosis of autoimmune diseases.

## 1 Introduction

Inborn errors of immunity (IEI) include a large, phenotypically and genotypically heterogeneous group of rare diseases caused by mutations in genes which play an important role in the development and functioning of the immune system (1, 2). One of the disease entities belonging to the IEI group is cytotoxic T lymphocyte–associated antigen-4 (CTLA4) insufficiency (3). It is typically inherited in an autosomal fashion (4). Physiologically, CTLA-4 is a checkpoint inhibitor expressed on T cells. CTLA-4, through competitive binding to CD80 or CD86, inhibits the connection of these molecules with the costimulatory signal receptor required for T cell activation (3, 5). CTLA-4 insufficiency, therefore, results in the impaired regulation of the T cell-dependent response and the development of symptoms resulting from autoimmunity (3, 5, 6). In the clinical picture of patients with CTLA-4 insufficiency, we can observe symptoms related to the hematopoietic system (pancytopenia and hypogammaglobulinemia, lymphoproliferation), respiratory infections, autoimmune endocrinopathies, and also to the digestive system (autoimmune hepatitis, primary sclerosing cholangitis, autoinflammatory intestinal diseases, celiac disease), dermatological diseases (psoriasis, atopic dermatitis) and rheumatic diseases (6–8). Most cases described in the literature show the first symptoms of the disease before the age of 18, but the phenotypic variability of patients (the occurrence of non-specific symptoms in different age groups) complicates and delays the diagnostic process (1, 2, 8). For this reason, the median age of patients at the time of diagnosis is estimated at approximately 26 years of age (7, 8). Most scientific reports on patients with CTLA-4 insufficiency concern adult patients. That is why it seems essential to analyze the phenotype and course of the disease in a pediatric patient. In this article we present the case of an almost 4-year-old patient with severe autoinflammatory symptoms who was treated with hematopoietic stem cell transplantation (HSCT).

## 2 Case description

A boy aged 3 years and 10 months was referred to the Department of Pediatric Hematology, Oncology and Transplantology due to thrombocytopenia (18 x 10^3^/μl). A week earlier, he had a mild upper respiratory tract infection, followed by a small-spotted hemorrhagic rash and skin bruising. So far, the child has developed normally. In the family, the child’s mother suffers from type I diabetes and autoimmune thyroiditis, while the mother’s sister suffers from celiac disease and Lenox-Gastaut syndrome. The physical examination revealed punctate petechiae on the skin, numerous hemorrhages and single petechiae on the soft palate. Additionally, bilaterally enlarged axillary lymph nodes were observed. Due to the clinical picture suggesting primary immune thrombocytopenia, the patient received an intravenous infusion of immunoglobulins (IVIG), after which moderate headaches and vomiting were observed. After 5 days of treatment, an increase in the platelet count was observed (245 x 10^3^/μl), and the patient was discharged home with recommendations for periodic monitoring at the hematology clinic.

11 months after the first admission, the patient was hospitalized again due to thrombocytopenia and anemia. The physical examination revealed features of atopic dermatitis, hemorrhagic ecchymosis and enlargement of the submandibular lymph nodes. In order to differentiate it from a malignant disease, a bone marrow biopsy was performed, which did not reveal any abnormalities. During hospitalization, the patient received an IVIG infusion, which resulted in an increase in the platelet count, but no change in hemoglobin (Hb) concentration. During the next 5 months, the patient was hospitalized three times due to thrombocytopenia flares. The treatment included IVIG infusions and dexamethasone.

**Figure 1:**
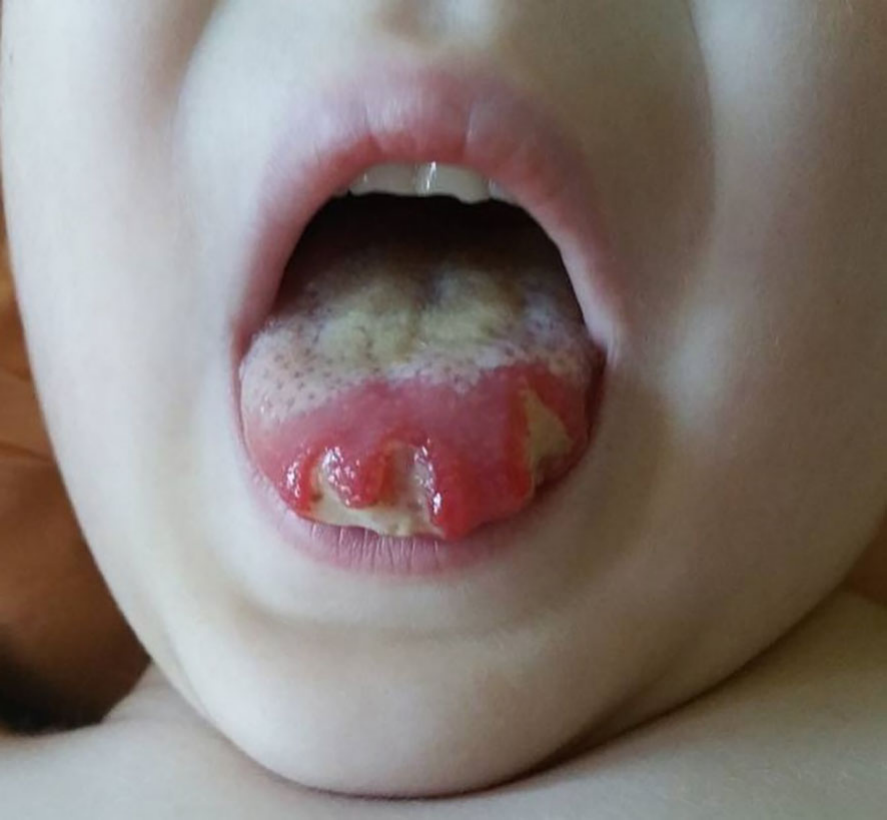
Tongue lateral border ulceration with surrounding erythema.

**Figure 2:**
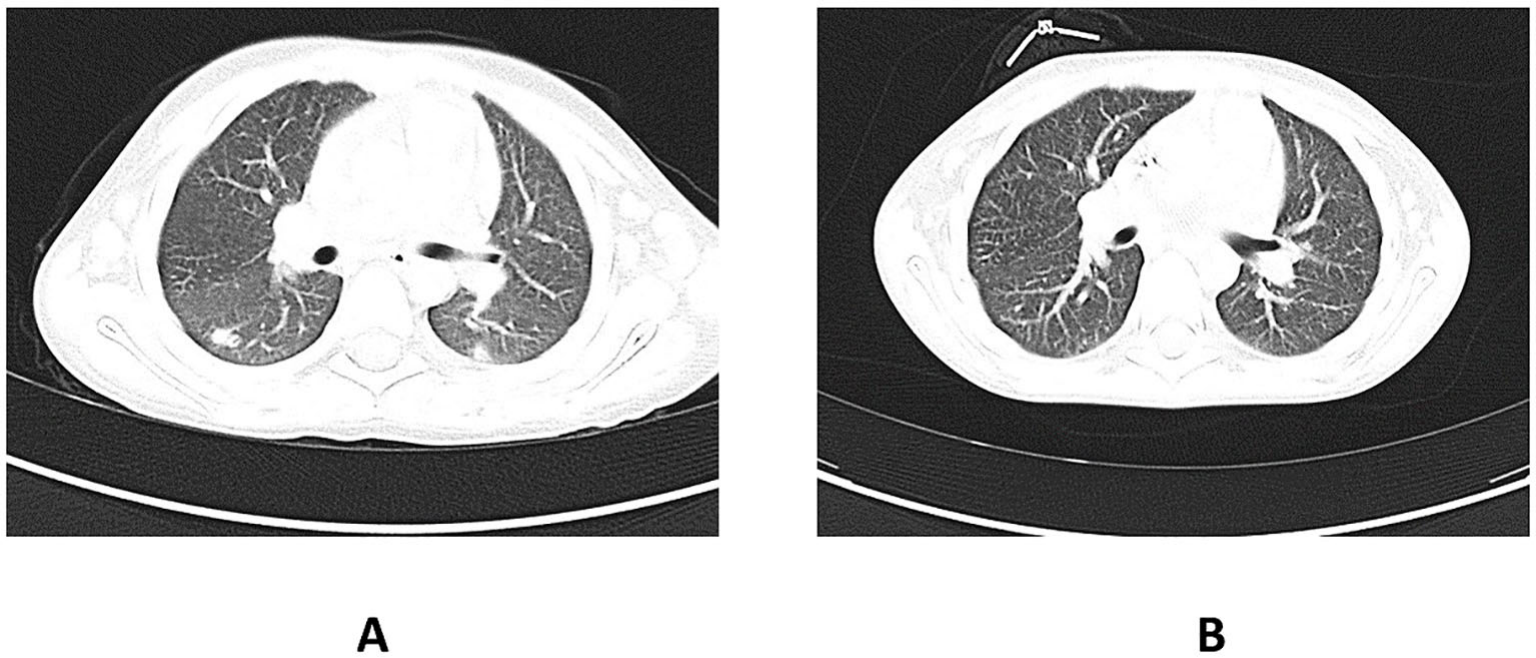
**(A)** shows the CT scan of the chest before the implementation of antifungal therapy in the patient. The pulmonary window shows nodular lesions in both lungs. **(B)** shows a CT scan of the chest taken one month after the initiation of the antifungal therapy. Visible regression of the changes described earlier. CT, computed tomography.

About 20 months after the first admission, the patient was admitted again due to agranulocytosis (neutrophils (NEU) – 0.0 x 10^3^/μl), anemia (Hb – 7.8 g/dl) and symptoms of gastrointestinal infection (diarrhea up to 20 times a day, fever up to 40°C). Intraoral examination revealed tongue lateral border ulceration with surrounding erythema (see **Figure 1**). Computed tomography scan showed bilateral lung lesions: nodular densities in segments 4 to 6 of the right lung and in segment 6 of the left lung, as well as areas of ground-glass shadowing in the posterior portions of segments 6 of both lungs. These changes were identified as probably fungal. A control CT scan performed one month after the implementation of antifungal treatment showed regression of the described changes (see **Figure 2**). In order to exclude proliferative disease, a bone marrow biopsy and trephine biopsy were performed again, which excluded the presence of cancer, but showed aplasia of the granulocytic system with an activated megakaryocytic system. Hemophagocytic syndrome (HLH) was also ruled out as the patient did not meet the diagnostic criteria. Additional tests revealed the presence of autoantibodies against: red blood cells, granulocytes, thyroid cells (anti-TPO; 146.1 U/ml) (without clinical indicators of gland function disorders) and anti-glutamic acid decarboxylase (GAD) autoantibodies (>250.0 IU/ml). The material for determining the concentrations of antibodies was collected 4 days before IVIG administration, which limits the possibility of the passive transfer of exogenous antibodies via IVIG. In the samples taken during endoscopic examinations of the gastrointestinal tract, inflammatory infiltrates were found, which may suggest an autoimmune disease. Based on the patient’s clinical picture, IEI with an autoimmune phenotype were suspected. Therefore, a genetic test was ordered. During hospitalization, the patient was initially treated with high doses of IVIG (1.0 g/kg for 5 days) and then with methylprednisolone (2 mg/kg). After initial improvement, the symptoms recurred.

During following weeks there was a significant, life-threatening deterioration in the patient’s clinical condition (NEU – 0.0 10^3^/μl, Hb – 9.1 g/dl). Despite a lack of confirmation of the genetic diagnosis, due to the rapid deterioration of the patient’s condition, the medical council decided to implement non-standard treatment. Therefore, immunoablative treatment using anti-thymocyte globulins (ATG) (according to the SAA program) was implemented. A good response was achieved (the number of granulocytes increased to 74 x 10^3^/μl), but after 2 weeks the symptoms recurred. On Day 24 after ATG administration, the white blood cells (WBC) count dropped to 0.67 x 10^3^/μl. Due to the initial good response to ATG treatment, it was decided to start immunosuppressive treatment in accordance with the program for the treatment of HLH (HLH 2004) - dexamethasone, etoposide and cyclosporine. Improvement was achieved (WBC – 6.27 x 10^3^/μl) after 4 weeks of treatment. Due to persistent hypogammaglobulinemia, the patient received IVIG infusions. Then, for 11 months, the treatment according to the HLH 2004 regimen was continued, but it was complicated by the symptoms of renal failure (urea – 86 mg/dl), elevated N-terminal pro b-type natriuretic peptide – 34 130 pg/ml without clinical symptoms of heart failure and cytomegalovirus infection treated with ganciclovir. After 2 weeks of treatment, the virus was undetectable.

The patient had a previously unreported heterozygous missense mutation in the *CTLA4* gene (c.356T>C) detected by the next generation sequencing (NGS) test, which was then confirmed by direct sequencing method. The mutation results in a change of leucine to proline at position 119. of the protein chain. The change was classified as disease causing by MutationTaster, deletorius by SIFT, and probably damaging by PolyPhen. The reference sequence NM_005214.4 was used for genetic testing. Due to the result of the genetic test, the patient’s parents underwent Sanger sequencing to determine whether the patient’s mutation occurred *de novo* or was inherited. In the patient’s mother, the same heterozygous variant of the *CTLA4* gene was detected. The literature describes a case of the patient with CTLA-4 deficiency caused by a missense mutation at c.356 position leading to the replacement of thymine by guanine, which means that arginine instead of leucine was located at position 119 (1). In our case, however, the mutation caused the substitution of thymine with cytosine, which in turn resulted in a change from leucine to proline at position 119. The description of the patient with CTLA-4 deficiency caused by a missense mutation at the same position (c.356) as in our case as well as the result of bioinformatic prediction combined with the information concerning our patient’s phenotype correlating with the genotype strongly suggest the diagnosis of CTLA-4 deficiency in our case, irrespective of a lack of functional tests, which could not be performed at that time due to their limited availability, but also because of the severe clinical condition of our patient requiring HSCT (7).

After receiving the genetic test result, due to a lack of availability of abatacept (which is a CTLA-4 fusion protein) at that time, a decision was made to qualify the patient for HSCT. Therefore, the patient continued treatment according to the HLH 2004 regimen until the initiation of conditioning before HSCT (according to Inborn Errors - Fludarabine 150 mg/m^2^, Treosulfan 42 g/m^2^) and graft-versus-host disease (GvHD) prophylaxis (cyclosporine 3 mg/kg/day). The patient underwent HSCT from a 9/10 unrelated donor with antigenic incompatibility at locus A 34 months after the first symptoms appeared. No early complications were observed during the procedure, and after the procedure, 100% chimerism of the donor cells was achieved.

**Figure 3:**
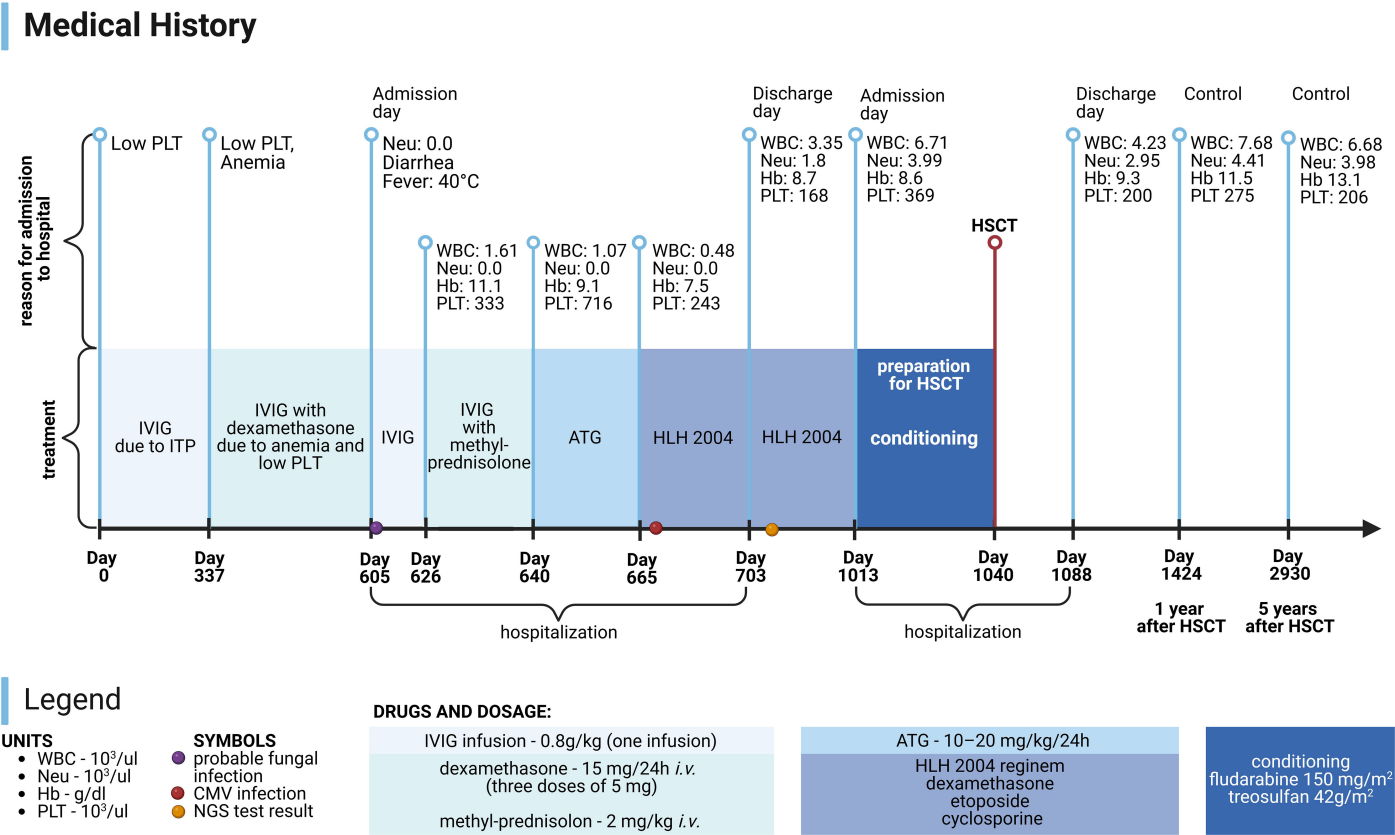
Medical History of patient. PLT, platelets; Neu, neutrophils; WBC, white blood cells; Hb, hemoglobin; HSCT, hematopoietic stem cell transplantation; IVIG, intravenous immunoglobulins; ITP, immune thrombocytopenic purpura; ATG, anti-thymocyte globulins; HLH 2004, treatment protocol for hemophagocytic lymphohistiocytosis; CMV, cytomegalovirus; NGS, next generation sequencing. Image created with biorender.com (accessed on 18 July 2024).

**Figure 4:**
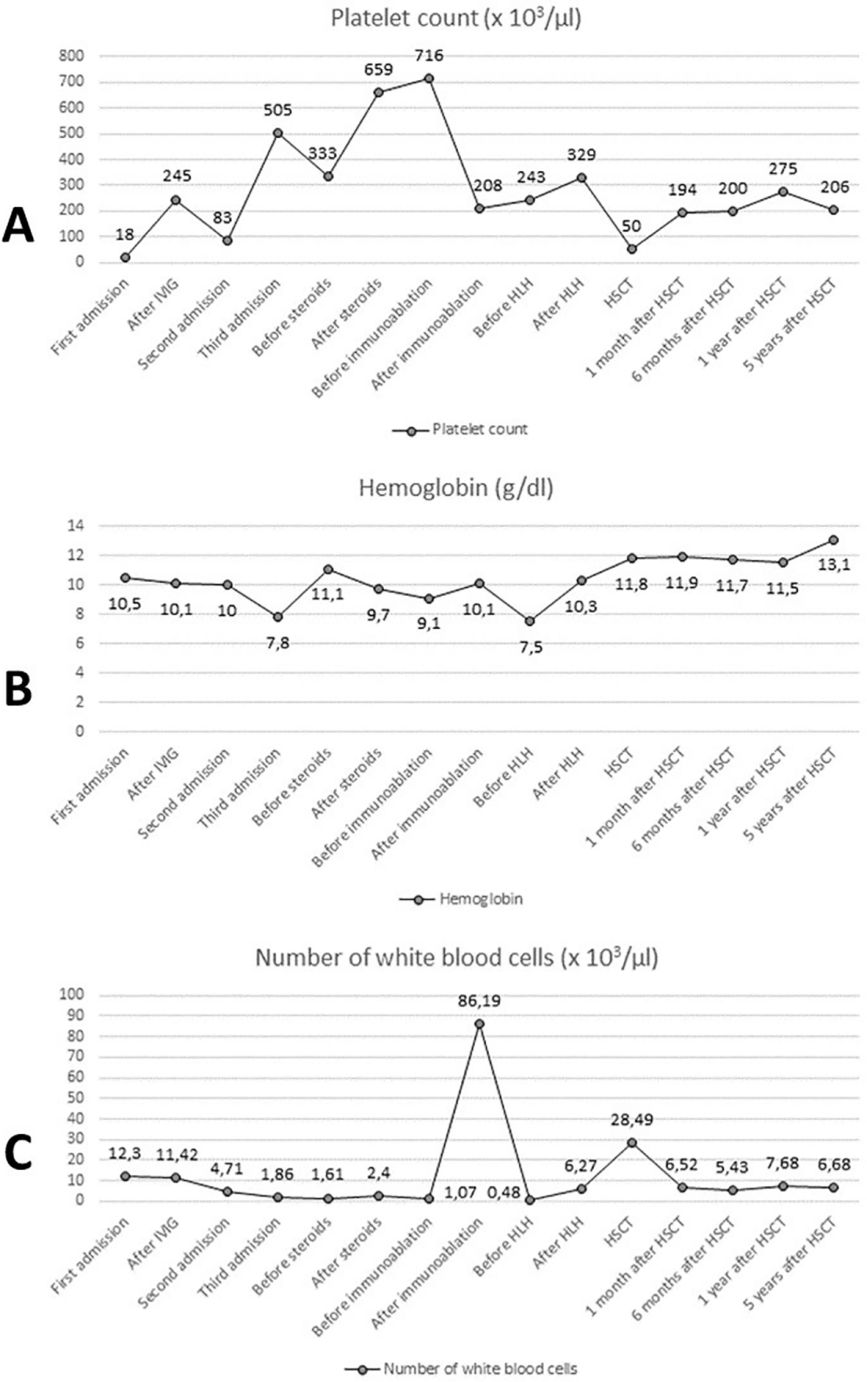
Laboratory test results: platelet count **(A)**, hemoglobin concentration **(B)** and number of white blood cells **(C)**. IVIG, intravenous immunoglobulins; HLH, treatment protocol (HLH 2004) for hemophagocytic lymphohistiocytosis; HSCT, hematopoietic stem cell transplantation.

Currently, the patient is alive and well, and his condition is stable. It has been 7 years since the genetic confirmation of the presence of a pathological variant of CTLA-4 (c.356T>C) and 6 years since the HSCT. The patient attends school and his physical development is normal (body weight: 40 kg - 41st percentile; height: 139 cm - 5th percentile, body mass index (BMI): 20.7 - 79th percentile). The patient remains under the care of the Immunology Clinic due to the post-HSCT follow-up. Medical History of the patient is presented in **Figure 3**. Laboratory test results of the patient are presented in **Figure 4**.

## 3 Discussion

CTLA-4 insufficiency was identified as the cause of immune dysregulation with the autoimmune lymphoproliferative immunodeficiencies phenotype in 2014 (7, 9). The clinical picture in mutation carriers is variable due to the incomplete penetrance of CTLA-4 insufficiency, estimated at approximately 67-71% (7, 8). Numerous infections of the upper and lower respiratory tract associated with hypogammaglobulinemia are the most common clinical manifestations of the disease in adults. Additionally, symptomatic patients may suffer from: pancytopenia, endocrinopathies resulting from autoimmunity, granulomatous lymphocytic interstitial lung disease, chronic autoimmune enteropathies, most often manifested by diarrhea, as well as rheumatic and dermatological diseases (atopic dermatitis, psoriasis, alopecia, vitiligo) (5, 7, 8). Some patients with CTLA-4 insufficiency have coexisting lymphoproliferation manifested by the presence of inflammatory infiltrates in the lymphoid system (spleen, lymph nodes, bone marrow), as well as in the lungs, gastrointestinal tract, central nervous system, skin, liver and kidneys (5, 7). Due to high phenotypic variability, symptomatic patients with CTLA-4 insufficiency may initially be diagnosed by physicians of various specialties, which is why it is necessary to constantly raise clinicians’ awareness of the wide range of IEI (8). There are few reports in the literature about the phenotype of pediatric patients with CTLA-4 insufficiency. Lanz et al. reported two cases of refractory CTLA-4 deficiency in pediatric patients. The first patient (P1) had been suffering from recurrent oral infections caused by Herpes simplex virus since childhood. At the age of 14, atopic dermatitis was added to the symptoms presented by P1. Additionally, the patient had frequent upper respiratory tract infections. After P1 turned 15, he was diagnosed with peripheral blood count disorders (neutropenia, thrombocytopenia, mild lymphopenia), as well as lymphadenopathy and splenomegaly. After excluding a malignant disease, P1 was tested for IEI, which led to the diagnosis of CTLA-4 deficiency. In P1’s mother, who had the same genetic variant, symptoms were limited to celiac disease and autoimmune thyroiditis. In the second case (P2), the first symptom of CTLA-4 deficiency were episodes of bloodless diarrhea occurring in P2 from the age of 8 and the celiac disease was diagnosed when P2 turned 10. However, despite the introduction of a gluten-free diet, no clinical improvement was achieved. Only the use of immunosuppressive treatment led to improvement in the clinical condition of P2; therefore, IEI was suspected (10). In our case, the patient had only sporadic respiratory infections, while the most clinically severe symptoms of the disease were thrombocytopenia with agranulocytosis and autoimmune enteropathy. Interestingly, despite the presence of anti-thyroid and anti-GAD antibodies, the patient did not develop clinically overt thyroid disease or celiac disease.

In the treatment of symptomatic cases of CTLA-4 insufficiency, IVIG infusions, steroid therapy, sirolimus, and abatacept are used. In the immediate control of respiratory tract infections, clinical improvement is achieved with IVIG infusions, which, in combination with steroids, are also used as first-line treatment for ITP (7). In order to control symptoms resulting from gastrointestinal involvement, the use of abatacept is recommended, which may also be effective in controlling cytopenia and maintaining it in remission (7). The effectiveness of pharmacological treatment in the symptomatic treatment of cytopenia is estimated at approximately 80%, but in our case, achieving lasting remission and control of the disease turned out to be impossible. The HSCT procedure used in the described patient, as a form of therapy for CTLA-4 insufficiency, is used in the case of autoimmune cytopenias which do not respond to pharmacological treatment or in patients with severe inflammatory infiltrates affecting systems other than the lymphoid system. In case of a severe disease, abatacept may be used as a bridge therapy to HSCT or as the treatment of choice if the patient is not qualified for HSCT (10). Although HSCT ensures permanent cure of the disease, it is rarely used due to a high risk of complications associated with the transplantation procedure (7). In the study by Tsifilis et al. evaluating the efficacy of HSCT as a form of therapy in CTLA-4 insufficiency in a group of 40 patients, the 3-year overall survival was 76.7% and disease-free survival was 74.4%. In the analyzed group, 7 patients died due to transplant related complications (grade IV acute GvHD, chronic GvHD and endothelial dysfunction/transplantation-associated thrombotic microangiopathy). One patient died due to diabetic ketoacidosis in full remission of the disease (11).

To sum up, in this case report we have described the case of a pediatric patient with severe CTLA-4 insufficiency, whose first symptoms of the disease appeared before the age of 4. The patient’s treatment with IVIG, steroid therapy and ATG did not bring the desired results (symptoms of autoimmunity recurred). Ultimately, the patient was successfully treated with HSCT.

## 4 Conclusions

The course of the disease described in the discussed case report expands our knowledge about the possible clinical phenotype of CTLA-4 insufficiency in a pediatric patient, and also indicates that due to the non-specific symptoms of diseases classified as IEI it is important to perform genetic tests before making a final diagnosis. An early diagnosis of the patient’s disease is extremely important because the treatment may be initiated before the disease symptoms become severe. Therefore, it seems essential that practicing pediatricians should take into account the possibility of CTLA-4 insufficiency in the differential diagnosis of autoimmune diseases. The patient whose case we have described in this report came to the clinic with the aforementioned symptoms only a year after the first description of CTLA4 insufficiency in the medical literature, so it is extremely important to constantly update the knowledge about IEI among practicing clinicians.

## 5 Patient perspective

The child’s serious illness was a difficult experience for his legal guardians. Seeing the child’s suffering, the parents felt helpless and extremely afraid for his life when the patient’s condition deteriorated significantly. Initially, they were looking for alternative methods of therapy using herbs of unknown composition that were recommended to them by a local healer. After making a genetic diagnosis and an unsuccessful attempt at pharmacological treatment in the face of increasing symptoms of autoimmunity, the parents were informed about the need to perform the risky HSCT procedure as it was the only chance to cure the child, and the parents gave their consent to it, hoping that the proposed treatment would help their child. By achieving clinical improvement, the patient returned to normal functioning and began to achieve further milestones. Due to significant weakness, the patient spent a long time in a lying position and had difficulty in walking. Currently, the patient is under the supervision of an immunological/hematological clinic and is pleased with the opportunity to have his case described in this article. Together with his legal guardians, the patient is delighted that the topic of CTLA-4 deficiency is being continually explored and hopes that it will contribute to raising awareness of the medical community about CTLA-4 insufficiency.

## Data Availability

The raw data supporting the conclusions of this article will be made available by the authors, without undue reservation.
